# The Input Matters: Assessing Cumulative Language Access in Deaf and Hard of Hearing Individuals and Populations

**DOI:** 10.3389/fpsyg.2020.01407

**Published:** 2020-06-19

**Authors:** Matthew L. Hall

**Affiliations:** Department of Communication Sciences and Disorders, Temple University, Philadelphia, PA, United States

**Keywords:** communication mode, deafness, early intervention, family language planning, language access profile, language assessment, sign language, speech-language pathology

## Abstract

Deaf and hard-of-hearing (DHH) children present several challenges to traditional methods of language assessment, and yet language assessment for this population is absolutely essential for optimizing their developmental potential. Whereas assessment often focuses on language outcomes, this Conceptual Analysis argues that assessing cumulative language *input* is critically important both in clinical work with DHH individuals and in research/public health contexts concerned with DHH populations. At the individual level, paying attention to the input (and the person’s access to it) is vital for discriminating disorder from delay, and for setting goals and strategies for reaching them. At the population level, understanding relationships between cumulative language input and resulting language outcomes is essential to the broader public health efforts aimed at identifying strategies to improve outcomes in DHH populations and to theoretical efforts to understand the role that language plays in child development. Unfortunately, several factors jointly result in DHH children’s input being under-described at both individual and population levels: for example, overly simplistic ways of classifying input, and the lack of tools for assessing input more thoroughly. To address these limitations, this Conceptual Analysis proposes a new way of characterizing a DHH child’s cumulative experience with input, and outlines the features that a tool would need to have in order to measure this alternative construct.

## Introduction

Deaf and hard-of-hearing (DHH) children present several challenges to traditional methods of language assessment, and yet language assessment for this population is absolutely essential for optimizing their developmental potential. The Joint Committee on Infant Hearing has been recommending routine and recurring language assessment for DHH children for at least the past 20 years (Joint Committee on Infant Hearing, 2000, 2019; Muse et al., 2013). In DHH populations, language assessment contributes to two important goals that can sometimes seem disconnected from one another: (1) optimizing the outcomes of an individual DHH child, and (2) optimizing the outcomes of the entire population from which the DHH child is sampled. This latter goal constitutes a public health objective, the achievement of which goes beyond any individual clinician’s responsibility. However, because public health data are typically aggregated over large corpora of individual assessment results, and those assessments are usually carried out by clinicians, the two are inextricably linked. This Conceptual Analysis argues that considering a DHH child’s language input is vital at both scales.

At the individual level, considering the child’s language input provides necessary context for understanding and interpreting assessment results. Language delays are common in DHH children, but for an individual child, these delays can either be unsurprising and (relatively) unimportant, unsurprising but important, or surprising and important (whether positive or negative). Characterizing the child’s cumulative experience with language input helps us differentiate these possibilities, and calls attention to recommendations that might otherwise be overlooked.

At the population level, it is essential to identify malleable factors that can optimize a child’s developmental potential so that evidence-based recommendations can be presented to future generations. For DHH children, the input that is in their environment throughout infancy and toddlerhood is a malleable factor of major importance; however, we still lack useful information about what kinds of early experiences with input are most likely to maximize language outcomes. A major reason for the absence of such information is the sheer complexity and diversity of DHH children’s experiences with linguistic input during the critical language-learning years of infancy and toddlerhood. This poses serious challenges for both clinicians and researchers, as explored below.

In clinical settings, time is precious. Although professional best practices encourage clinicians to take thorough language histories in early intervention contexts (American Speech-Language-Hearing Association [ASHA], 2008), the need to perform a diagnostic assessment may be considered a more urgent priority, especially if a child is participating in a program where assessment outcomes inform the child’s continuing eligibility for services, school placement, IFSP/IEP goals, etc. The amount of time devoted to gathering a language history may therefore be very limited, if one happens at all. And because education about the importance of gathering language histories is often provided with respect to multilingual populations (e.g., American Speech-Language-Hearing Association [ASHA], 2010), the clinician may not believe that collecting a thorough, cumulative history from a monolingual family is worth the time. However, even DHH children from monolingual homes have considerably diverse experiences with language input, as the following section on “language exposure” vs. “language access” will explain.

A second significant problem is that even if clinicians are committed to collecting comprehensive data about a DHH child’s cumulative experience with linguistic input, they have no empirically tested tools with which to do so. The only formal, research-based tools that are presently available are all developed for multilingual children from hearing families (e.g., LEAP-Q, Marian et al., 2007; BESA, Peña et al., 2018; LEAT, DeAnda et al., 2016, inter al.). Although such tools offer useful frameworks for thinking about input, they would need careful adaptation before becoming suitable for use with DHH populations. But until the need for such tools is more widely appreciated, there is little incentive for them to be developed or used.

In the meantime, clinicians may use informal assessments/interviews, and may even have the opportunity to observe the child’s current input in naturalistic settings. But this raises a third problem: using observational language samples to understand the nature of a child’s input is only a valid approach when both the input and the child’s access to it have remained fairly constant throughout the child’s life. For DHH children, neither can be assumed: children’s auditory access to spoken language often changes over time, as does their interlocutors’ use of and proficiency in various forms of manual communication. Thus, strategies that serve SLPs well when working with hearing children often do not suffice for clinical work with DHH children. Current technology offers no easy solutions: no automated tools for characterizing visual input are available or even on the horizon; nor would their sudden appearance allow for a cumulative history to be obtained. As a result of this constellation of factors, assessment of DHH children’s cumulative experience with linguistic input is often limited in clinical contexts, despite the well-established understanding that language input plays a pivotal role in language acquisition.

In the research literature, one strategy has been to rely on recent advances in technology such as Language ENvironment Analysis (LENA) software, which records and to some extent categorizes the auditory input in a child’s environment. In DHH populations, this approach is becoming more common as a way of linking a child’s language outcomes to their language input (e.g., Aragon and Yoshinaga-Itano, 2012; Van Dam et al., 2012; Wiggin et al., 2012; Suskind et al., 2013, 2016; Ambrose et al., 2014, 2015; Sacks et al., 2014; Vohr et al., 2014). However, it is imperative to understand that LENA systems are inherently limited in the insights that they can offer. First, they provide no data at all about the child’s experience before they received and began using a LENA system. Thus, unless a LENA device has been used since birth, there is no way for this information to provide information about a child’s cumulative history of linguistic input. Second, LENA systems provide information about auditory input only: they are entirely insensitive to any form of visual communication. Accordingly, LENA systems have no way to differentiate spoken input that is produced without manual accompaniments from spoken input that is accompanied by either signs or cues (which would also not be distinguished from one another). LENA systems would also interpret periods of silence as the absence of input, even if a sign language were being used. Thus, LENA systems are wholly incapable of assessing a DHH child’s experience with non-auditory forms of input, which in turn precludes any progress in understanding how difference experiences with such forms of input relate to subsequent language outcomes. Third, a LENA system knows only what *it* hears, which is not the same as what a DHH *child* hears. In order for LENA data to be a valid representation of the child’s auditory access, a separate process would need to be implemented that links the LENA recording to datalogging from a child’s hearing technology, which is itself only an approximation and insensitive to the extent of a child’s residual hearing. Moreover, even if LENA data were appropriately integrated with datalogging and adjusted for child-specific hearing profiles, the former two problems remain. Thus, although LENA data can illuminate some aspects of the relationship between language input and language outcomes, they cannot document a child’s cumulative history of access to various types of input.

A second response to the complexity and diversity of DHH children’s cumulative experiences with input has been to rely on the construct of “communication mode” as a proxy for describing DHH children’s cumulative experience with language input. Unfortunately, this construct is typically used in ways that are too simplistic to reflect children’s actual experiences, and too variable across studies to support meaningful generalization (Hall and Dills, 2020). This Conceptual Analysis argues that clinicians and researchers must reconsider the ways that we assess DHH children’s input, adopting methods that recognize its diverse and multidimensional nature throughout the crucial language-learning years of infancy and toddlerhood, and take dose-response functions into consideration.

This manuscript provides only a high-level conceptual overview of what the alternative construct should look like; the primary goal is to underscore the importance of routinely collecting information about DHH children’s cumulative experience with language input, not just their language outcomes (or current experience with input), when performing language assessment.

### Language Exposure vs. Language Access

Before proceeding, it is necessary to introduce a conceptual distinction that may be new to some readers, particularly those who do not regularly work with or think about DHH populations: namely, the distinction between language *exposure* and language *access*. No child ever learns a language that they are not exposed to. But for DHH populations, language *exposure* (i.e., the presence of input in the child’s environment) is not enough. What is necessary is *access*: that is, the child must be able to perceptually receive and cognitively process the signals that are being sent. This distinction is not a new one; Moeller and Tomblin (2015) refer to this distinction as “language input” versus “language experience,” and Harris (2013) distinguishes “language input” versus “language uptake.” Despite the variations in terminology, the core idea is that for DHH children, it is not enough to simply consider what kinds of linguistic signals are being *sent to* a child. Instead, it is necessary to think about the linguistic signals that that child is *receiving*. Moeller and Tomblin (2015) identify several factors that influence a DHH child’s auditory access to spoken input: aided audibility (including appropriate fitting of hearing aids and mapping of cochlear implants), consistent use of hearing technology, and the nature of the linguistic input in the child’s environment (quantity and quality). This model can easily be extended to encompass visual forms of communication as well, which is perceptually accessible to DHH children without technology (except in children who also have reduced vision). For the remainder of this paper, unless otherwise noted, the term “input” should be understood as referring to all and only those linguistic signals to which a child has access: whether auditory or visual. Note that it is also possible for a DHH child to have only limited access to any linguistic input; indeed, it is this state of having limited access to input (rather than deafness itself) that creates developmental risk (Hall et al., 2019). More attention is given to this notion of limited access at the population level; first, we consider the importance of assessing cumulative experience with input at the individual level.

### Language Input Matters at the Individual Level

#### Discriminating Disorder From Delay/Difference

At the group level, language skills in DHH children are often found to be, on average, between 1 and 2 standard deviations below those of test norms (which almost invariably represent monolingual children with typical hearing) or demographically matched hearing controls (Koehlinger et al., 2013; Tobey et al., 2013; Ambrose et al., 2014, 2015; Tomblin et al., 2015; Eisenberg et al., 2016; Geers et al., 2017; Lewis et al., 2017; Hoffman et al., 2018; Lederberg et al., 2019; Antia et al., 2020). Of course, there is considerable heterogeneity at the individual level, and clinicians are charged with supporting one child at a time: assessing their current level of proficiency, making informed inferences about the reasons behind specific areas of weakness or strength, devising individualized interventions, and making recommendations to the child’s family and the other allied professionals on the child’s team. In the all-too-common event that a child shows language skills that are far behind their chronological age and cognitive potential, one important question is whether this represents a delay/difference or a true language disorder^1^. One way to address that question is to consider growth over time; if the child is making one year’s worth of progress in one year’s time, then there is little concern about a language disorder. However, this approach requires the passage of time, which is a precious resource in early childhood. Dynamic assessment (Gutiérrez-Clellen and Peña, 2001) is an alternative that is commonly practiced with culturally- and linguistically diverse populations (e.g., Rosemary et al., 1996; Gillam Ronald and Peña Elizabeth, 2004), but has not yet been widely adopted for DHH populations, despite calls to do so (Mann et al., 2014). A third and also-underutilized strategy for discriminating disorder from delay/difference is to consider the child’s input. Doing so helps reveal whether the observed outcomes are unsurprising and unimportant, unsurprising but important, or surprising and important.

##### Unsurprising and unimportant

Hearing children who are successfully acquiring more than one language often appear to score lower on language-specific assessments or to meet language-specific milestones later than their monolingual peers (e.g., Hoff et al., 2012). However, it is now widely understood that such putative differences may in fact be epiphenomenal: reflecting weaknesses in a tool’s ability to gauge a multilingual child’s true developmental state, rather than revealing a meaningful problem (e.g., Pearson et al., 1993). What makes this situation of little concern is evidence that the child’s knowledge in the two (or more) languages is complementary and mutually reinforcing, together with evidence that the child is meeting the kinds of milestones that are not language-specific (e.g., increasing MLU, turn-taking, fast mapping, etc.). Such a child is likely to develop age-appropriate command of both languages prior to school entry.

Deaf and hard-of-hearing children may fall into this category if they have had good perceptual access to multiple languages (spoken or signed), such that the primary reason that they fall behind monolingual norms in one language is because they also have knowledge in another language that is not being credited. Unfortunately, this situation is uncommon; language delays in DHH children are much more likely to fall into the next category.

##### Unsurprising but important

Deaf and hard-of-hearing children often have reduced access to their primary language (spoken or signed) without also having access to another language. If a child has had very little access to input in a given language, then it is unsurprising to find that they are not acquiring that language as a monolingual would. But if this is the case in the child’s strongest (or only) language, then the delay becomes highly important, even if its cause is unsurprising. Unlike children with access to multiple languages, it cannot be assumed that children with reduced access to one language will catch up to their typically developing peers, and the delays that they experience are not an epiphenomenon of having knowledge distributed across multiple languages. Instead, these are true delays that have true consequences, such as arriving at kindergarten without the skills needed to succeed (Hall et al., 2019).

In this case, the most straightforward approach to intervention would be to consider how to most effectively increase the child’s access to input. This might include attempting to alter the child’s perceptual access to the input around them, altering the input around them to be more accessible given the child’s perceptual abilities, or both. To determine the most effective course(s) of action, it is important to identify the most significant barriers that have been limiting the child’s access to input to date. A child with a late-identified hearing loss may simply need effective amplification. A child who has bilateral cochlear implants but only wears them inconsistently might benefit from parent counseling about strategies for increasing device use. For a child with no auditory nerve whose family refuses to use visual communication, a different kind of counseling is in order. In all cases, it is appropriate to consider what growth rate would be needed in order to achieve age-appropriate skills by school entry, and whether that growth rate is realistically attainable under the current course of action. If the answer is no, it is appropriate to consider whether there may be other courses of action worth pursuing.

##### Surprising and important (negative)

Although most DHH children experience reduced access to input to some degree, not all will fall into the situation described above, where their cumulative access to input has been so limited that it is an immediate red flag for intervention. A child for whom English has constituted 10% of their input would certainly fall into that category, but not a child whose input has been 90% English. But this raises an important question: at what point should reduced proficiency no longer be attributed to reduced access to input? In other words, how much access to a given language does a child need to have before we are surprised to find that they are not acquiring it?

Although there is surely no hard-and-fast answer to this question, research in hearing multilinguals is beginning to find that when 60% or more of a child’s cumulative input has been in Language A, standard scores from monolingual norms can be used without increasing the risk of falsely diagnosing a disorder (Cattani et al., 2014). These findings suggest that if a child scores below the average range despite 60% or more of their input consisting of access to Language A, clinicians are justified in suspecting that something is amiss, and that it is more than can be attributable to reduced access to Language A.

The next question at that point is whether the locus of the problem is within the child’s mind or in the child’s environment. Here is where dynamic assessment using a range of communication methods is most useful. If the child shows little modifiability across any type of input (signed or spoken), a language disorder may be indicated, and language therapy can be designed accordingly. If, however, the child is responsive to some types of communication, then a language disorder is unlikely and a shift in communication strategy may be warranted.

##### Surprising and important (positive)

There is another type of surprising and important finding: this time in a positive direction. A child may show surprisingly good command of a language that has constituted only a small proportion of their cumulative experience. This might be revealed through dynamic assessment as described above, but it would ordinarily be missed through static assessment that does not thoroughly characterize the child’s cumulative experience with linguistic input. In many cases, only the child’s strongest language is assessed; in contrast, input-informed assessment involves evaluating all of the languages that the child is acquiring and considering the observed degree of proficiency in relation to their prevalence in the child’s input.

For instance, suppose a child is evaluated in two languages, with a standard score of 80 in Language A and 70 in Language B.^2^ A typical outcomes-focused approach to assessment would likely note that the child is doing better in Language A and might recommend prioritizing that language on the assumption that the child has a smaller gap to close there, and that it will accordingly be easier to do so. However, it is not necessarily valid to assume that a smaller gap will be easier to close. Suppose we learn that the child’s input to date has consisted of 70% Language A and 30% Language B. In this case, the child’s proficiency in Language B is both surprising (given how little access there has been to date) and important (in that the child might actually have an easier time closing the gap if more of their input were in Language B). If the child’s scores were equivalent in both languages, this would be even more clear – but only if the clinician had information about the child’s cumulative experience with linguistic input.

#### Setting and Tracking Input-Related Goals

In the United States, families of DHH infants and toddlers typically receive early intervention services as part of an Individualized Family Service Plan under Part C of the Individuals with Disabilities Education Act. These plans involve setting specific and measurable goals, most of which are functional in nature (e.g., “Adrian will say which snack he prefers using spoken words.”). To facilitate the child’s progress toward (or past) these goals, professionals on the IFSP team may include additional goals. For example, where language outcomes are concerned, a speech-language pathologist will likely have identified certain outcome goals that the child is working toward (e.g., more utterances with an MLU > 4, clearer articulation of fricatives, more conversational turns, etc.), and will routinely monitor the child’s progress toward those outcomes. However, it can often be valuable to set goals related to the child’s *input*. For example, if the child/family struggles with consistent use of hearing technology, a goal might be that the child keep their hearing aids on and working for no less than 8 hours every day. This goal can be tracked using datalogging from the child’s hearing devices, but other goals require different approaches to measurement. For example, consider a child who is acquiring more than one spoken language. The SLP may realize or suspect that the family is not providing sufficient input in one language or the other to support the child’s acquisition of both, and so might set a goal that for the next 6 months, the child’s input consist of nothing less balanced than a 60–40% split, or might recommend creating a family language plan in which the lesser-used language is boosted to at least 3 hours a day. The SLP can then gather information about whether this goal is being met, whether through naturalistic observation, collecting language samples, administering surveys about language use, or conducting conversational interviews with the child’s caregivers. The same can be true if, for instance, a family intends their DHH child to become a proficient user of some form of manual communication. If that goal is to be achieved, the child will need to have appreciable amounts of input in that type of communication, and it will need to be tracked across all of the contexts in which the child spends significant amounts of time. Having this information is helpful whether the child is making good progress toward their outcome goals or not. If they are, the family may be informed that their efforts are paying off and be encouraged to maintain their effort. Or, if the family had decided to limit use of their home language in order to support the child’s eventual language of education, they might benefit from knowing that their child is doing well enough in the dominant language that they can start using their home language more without compromising the child’s success. And if a child is struggling, it is essential to know what the cumulative input has been like in order to determine whether this outcome is unsurprising and unimportant, unsurprising but important, or surprising and important, as discussed above. In the absence of information about the child’s cumulative experience with language input, appropriately setting and tracking goals becomes much more difficult.

## Language Input Matters at a Population Level

Despite many cases that would be considered successes at the clinical level (i.e., one child at a time), DHH children as a population remain at serious risk of not developing age-appropriate proficiency in any language by the time they enter school. The lack of true population-based datasets in the United States makes it difficult to know for certain, but large, multi-site/multi-state studies such as CDaCI, OCHL, and NECAP typically report language outcomes in DHH children that are 1–2 standard deviations below their hearing peers, or language quotients below the 80% threshold (Koehlinger et al., 2013; Tobey et al., 2013; Ambrose et al., 2014, 2015; Tomblin et al., 2015; Eisenberg et al., 2016; Geers et al., 2017; Lewis et al., 2017; Hoffman et al., 2018; Yoshinaga-Itano et al., 2018). A separate and more recent study of over 336 DHH children between kindergarten and second grade reported similar outcomes on measures of spoken language, with mean scores again ranging from 1 to more than 2 standard deviations below the normative mean (Lederberg et al., 2019; Antia et al., 2020). These values are commensurate with the findings of a large, longitudinal, population-based study in Australia (LOCHI; see Ching et al., 2010, 2018, for language outcomes at age 3 and 5, respectively). Equally concerning are recent findings from Norway (Wie et al., 2020), where all but two of the deaf children who received early, simultaneous, bilateral cochlear implants were followed from implantation through elementary school. Although not a large n, the data represent virtually the entire population. These were children who had no additional disabilities and received early intervention services focusing on spoken language acquisition, and therefore represent the most optimistic outcomes scenario. The authors reported that although these deaf children appeared to be closing the gap with their hearing peers as they approached school entry, gaps in receptive vocabulary and expressive grammar reappeared and remained present for the duration of the observation period (up to 6 years post-implantation). Outcomes such as these suggest that roughly half of DHH children with bilateral hearing loss^3^ -even those without additional diagnoses that might impede language acquisition- are not developing age-appropriate language skills. Clearly, the *status quo* is not allowing DHH children as a group to flourish. Indeed, even those DHH children who score above the 16th percentile are likely underperforming their true potential.

To those who are accustomed to working with individual children, especially in clinical contexts, it may be tempting to apply the same standards of success to populations. However, to do so is to make a serious mistake. In clinical assessment, it is commonly and correctly understood that although a population may be defined as having a certain expected score on average (e.g., standardized assessments), any individual sampled from the population may deviate from that score to a certain extent without raising suspicion that they may in fact have been sampled from an atypical distribution. The extent of this allowable deviation is commonly termed “the average range,” and although conventions vary by discipline and instrument, plus or minus one standard deviation is a common enough criterion that it will suffice to illustrate the present point. It is perfectly reasonable to be fairly unconcerned about an individual who scores an 86 on a standardized assessment where the mean is 100 and standard deviation is 15. However, if the mean of a sample of many individuals is found to be at 86, then that population is evidencing major deviation from expectations. The reason for this seeming double standard is the Central Limit Theorem, according to which the mean of a sample will converge on the mean of the population from which it is drawn as the sample size increases (specifically, in proportion to the square root of the sample size). Therefore, if a sample contains 100 individuals, the “average range” for the mean of that sample is no longer 85 to 115; rather, for a two-tailed test at alpha = 0.05, it would be from a lower bound of 97.06 [i.e., 100–1.96^∗^(15/sqrt(100)] to an upper bound of 102.94 [i.e., 100 + 1.96^∗^(15/sqrt(100)]. This is precisely equivalent to a *z*-test: comparing a sample against a population distribution where the mean and standard deviation are known. Finding that the sample mean falls outside the expected range of variation licenses the inference that the population from where the sample was drawn has a different mean than the reference population; however, this only becomes meaningful if the magnitude of the deviation (i.e., the effect size) is also large. In the case of a sample mean of 86, the mean would be shifted downward by nearly one full standard deviation. Assuming that the sample distribution is normally distributed, this means that roughly 50% of the sample (and, by inference, the population from which it was drawn) would fall below the clinically defined boundaries of the “average range” for individuals. For comparison, only about 16% of individuals in the reference population would be expected to score in that range: a risk ratio of 50/16 = 3.125, which equates to a 212.5% increase in risk relative to the reference population. Unfortunately, such scores are sometimes taken as evidence of success in studies of language outcomes in DHH children (e.g., Wie et al., 2020), rather than evidence that major disparities persist.

The search for ways to better support DHH children continues. As of this writing, the American Centers for Disease Control and Prevention have issued a call for proposals in response to the need for better monitoring of language outcomes and other developmental progress in DHH children after the initial processes of hearing screening, audiological diagnosis, and referral to/enrollment in early intervention. This call draws particular attention to how little is currently known about practices that will optimize DHH children’s developmental potential:

“While collaborative efforts by CDC, states, and other partners have helped lead to the early identification of thousands of children who are D/HH each year, their developmental and language outcomes are often unknown, and these data are not routinely collected by CDC or state EHDI programs. Furthermore, it is currently unclear what actions beyond early identification should be taken by public health to help reduce adverse consequences of hearing loss and ensure that children who are D/HH are ready for success in early childhood” (Centers for Disease Control, 2020).

The call goes on to identify the key role that assessment plays in filling these knowledge gaps:

“The current lack of public health capacity to document and assess the intervention services and associated outcomes of early-identified children who are D/HH at the state and national level makes it challenging to:

• Assess the developmental progress to ensure all children who are D/HH are achieving age-appropriate milestones and are ready for success in early childhood;
• Identify strategies, in addition to those beyond early identification, to help assess and reduce adverse consequences of hearing loss;
• Assess and document the success and impact of EHDI activities across the United States” (Centers for Disease Control, 2020).

In particular, this second goal of identifying strategies to reduce the adverse consequences of hearing loss would be easier if we knew more about DHH children’s cumulative experiences with linguistic input. Delayed or incomplete mastery of a first language is one of the most serious adverse outcomes that DHH children face. Although many factors influence language acquisition, the input itself is surely among the most crucial. There may be no guarantee that a child will successfully acquire a language that is present in their input, but if they lack sufficient access to a given language, we can be absolutely sure that they will *not* acquire it.

There has been no shortage of attempts to identify what kinds of early experiences with linguistic input are most likely to yield subsequent language mastery (for recent reviews, see Belzner and Seal, 2009; Fitzpatrick et al., 2016; Erbasi et al., 2017; Demers and Bergeron, 2019). However, these efforts have largely failed to yield consensus, for several reasons. First, there has been disagreement over whether success should be understood as mastery of a *spoken* language, mastery of *at least one* language, or achieving the goals that matter to the child’s parents, even if those goals represent less than the child’s full potential.^4^ The extant research has almost exclusively adopted spoken language acquisition as the barometer of success; therefore, very little is known about the factors that support successful acquisition of a sign language by children who are not among the ∼5% born to parents who are already proficient signers. Second, even when looking only at spoken language outcomes, the available results are highly mixed and based on studies of low methodological quality (Fitzpatrick et al., 2016; Demers and Bergeron, 2019). Third, and most relevant to the present argument, the very *construct* that researchers have used in an attempt to answer this question (i.e., “communication mode”) is ill-defined. Hall and Dills (2020) point out that in addition to the absence of any uniform operationalization of the term, it typically does not provide any information about what a child’s experience was like during infancy and toddlerhood, and it commonly conflates types of input that are very different (e.g., ASL, sign-supported speech, and manually coded English). They identify the desiderata of a better alternative and argue that until such an alternative is available, it will remain impossible to identify the kinds of strategies that the CDC rightly identifies as crucial gaps in knowledge. A high-level conceptual overview of what this new method might look like is provided below (readers interested in a more applied introduction are referred to De Anda and Hall, in prep). However, the primary goal of this section is merely to make the point that if the goal is to identify strategies for improving outcomes, then assessing outcomes alone is insufficient: assessing the input is also necessary. This section further argues that in order to be maximally useful at the population level, measures of input should support bottom-up grouping strategies, and allow exploration of dose-response relationships between language input and language outcomes.

### Language Input as an Upstream Determinant of Language Outcomes

At the 2020 Early Hearing Detection and Intervention conference, keynote speaker Dr. Michael Warren (Associate Administrator of the United States Maternal Child and Health Bureau) emphasized the importance of identifying upstream causes of later outcomes. He argued that intervening on upstream factors is a more efficient and more effective approach to public health than attempting to treat problems that arise downstream. Given that language input is necessarily antecedent to language outcomes, efforts aimed at improving language outcomes should pay close attention to language input: particularly to input during infancy and toddlerhood, when the human brain acquires language most readily. However, given the aforementioned limitations of communication mode as a construct, it is worth considering the desiderata of a better measure of language input for DHH children. The following recommendations are drawn from Hall and Dills (2020).

First and foremost, a useful measure of language input should have a clear and consistently applied operational definition. This is a prerequisite for establishing generalizability across studies.

It should capture a child’s cumulative experience with linguistic input over a given time window of interest. Ideally, this window would be prior to the point at which outcomes are being evaluated. There is a danger in measuring outcomes as a function of the child’s *current* input, since their current situation may be a result of their language proficiency rather than a cause of it. Again, the ultimate goal of population-level outcomes is to identify upstream predictors that can inform recommendations for future generations.

A useful measure of language input should have a way to represent the extent to which a child has had limited access to linguistic input, whether it be because of late identification, delayed availability or inconsistent use of effective hearing technology, delayed onset or infrequent use of visual communication, etc. While many of these reasons may be theoretically preventable, their impact (or lack thereof) on a child’s experience is still relevant for understanding that individual child’s outcomes, and must be included as part of the construct. Counter-intuitive though it may seem, the necessity of including something like a “limited access” category as part of a child’s input can be appreciated by considering two children whose environment consists of nothing but spoken English, of whom one gained excellent auditory access to spoken language at 9 months and the other at 27 months. Without including “limited access” as an input category, both children would appear to have 100% English. Including a “limited access” category reveals that the first child’s experience has been 75% English, 25% Limited Access, while the second child has had 75% Limited Access, 25% English. Clearly, the inclusion of this category results in a more faithful representation of their experience.

An existing construct like “hearing age” would likely share variance with a measure of “limited access” for some but crucially not all children. First, “hearing age” measures the time that has elapsed since the onset of auditory access; it does not capture factors that describe the *extent of access* during that time (e.g., appropriateness of fitting/mapping, consistency of device use, and listening environment). Second, “hearing age” would only be a valid proxy for “limited access” among children who did not have access to visual communication prior to the onset of auditory access. For example, consider another hypothetical child whose family began using sign-supported speech as soon as the child referred on their newborn hearing screening, and then switched to spoken English without sign when the child’s cochlear implants were activated at 9 months. By 36 months, this child’s experience of auditory access to English will be the same as that of the previous child who was also activated at 9 months; however, this child would have 0% Limited Access (and 25% sign-supported speech instead).

Similarly, a construct like “age of acquisition” (more commonly used with respect to sign languages) has comparable limitations: it identifies only the point at which access to a sign language began, but provides no information about how much experience the child then had with signed input. Likewise, it provides no information about the extent to which a child did or did not have auditory access to spoken language prior to (and after) the onset of signing. Thus, the measure of “limited access” would need to be sensitive to all of these considerations.

A useful measure of language input must make distinctions among types of communicative systems that are fundamentally different. For example, cued speech provides phonological information that helps to disambiguate words that look alike while speechreading. Manually coded English systems emphasize morphosyntax by pairing every spoken morpheme with a signed equivalent. Distinct from both of those is a broader category often called “sign-supported speech.” Like the previous two, the utterances in such a system are generated by the grammar of a spoken language (e.g., English). But unlike cued speech, the manual components of this signal have semantic content. And unlike manually coded English, the manual components do not include inflectional or derivational morphemes; often, there are no function words at all. Instead, this type of communication generally involves strings of signs that correspond to selected content words in linear order. This category encompasses practices that include Conceptually Accurate Signed English, simultaneous communication, “total communication” (misnomer though it may be), and baby sign. There may certainly be value in distinguishing among these subtypes of communication; however, distinguishing sign-supported speech from manually coded English and cued speech would be a good first step in the right direction.

Even more importantly, a useful measure of language input would distinguish natural sign languages from the types of communication described in the preceding paragraph. Unlike all of those, utterances produced in a natural sign language are not generated by the grammar of a spoken language. The fact that sign languages have their own grammars seems widely recognized when describing communication options to parents, but it somehow seems to be forgotten when interpreting research that fails to distinguish natural sign languages from other forms of manual communication.

A construct of this nature would be better able to reflect the actual experiences of DHH children than the currently dominant approach of simply identifying a child’s “communication mode.” Although the examples given above have been purposely simplistic for the sake of convenience, a construct that had the above-described properties would be able to describe more realistic profiles: for example, a child whose input by 36 months has consisted of 40% limited access, 20% English without sign, 15% sign-supported speech, and 5% ASL. Another child might have 40% limited access, and 60% English without sign. Still another might have 10% limited access, 30% Spanish, 30% English, 15% cued Spanish, and 15% cued English. Although it is hopefully now clear how this information is clinically useful at the individual level, such heterogeneity presents challenges to researchers working at a population level, who need either categorical or continuous variables to use as predictors. The constructs described above are perfectly capable of generating continuous values for a predictor variable that focuses on one type of input at a time; however, the argument here is that such an approach might be misleading, in that putative effects of variation in one category may in fact by epiphenomena of changes in another category, since this construct is fundamentally compositional in nature. It is argued that a better approach is to develop a categorical variable whose values represent various combinations of experiences. In this way, a child’s complex experience can still be represented with a single categorical value, since that value itself describes a multidimensional experience. A strategy for achieving this is described below.

### Top-Down vs. Bottom-Up Grouping

Historically, research on DHH children’s experience with linguistic input has involved top-down grouping strategies. That is, a researcher or policy maker makes a set of *a priori* decisions about what groups are relevant to compare, sets criteria for inclusion in those groups, and then proceeds to compare outcomes between/among those groups. Usually, this involves comparing a DHH children who use listening and spoken language exclusively against those who do not (Hall and Dills, 2020). One virtue of this approach is that it covers the entire parameter space, since every child can be characterized as belonging to either one or the other. According to recent data from the National Center for Hearing Assessment and Management [NCHAM] (n.d.) in the United States, this division also results in roughly equal-sized groups: 49% of the 303 families who responded to the survey reporting using listening and spoken language (LSL) exclusively, and 51% did not. However, the 51% reported a diverse set of experiences, including mostly LSL with some signs or cues (17%), roughly equal amount of signed and spoken communication (14%), mostly cued speech (12%), mostly signing with some speech (3%), sign language only (3%), and other (1%). Treating these children as if they all had the same experience with language input precludes the possibility of discovering subsets of children within this group that might have stronger language outcomes than others.

It may be tempting at this point to propose that a better solution might be to simply divide the 51% into smaller groups like those listed above; however, this too has problems, as noted above. Rather than attempting to refine the top-down categories, a better solution may be to abandon them entirely, in favor of bottom-up, data-driven grouping strategies in which DHH children’s idiosyncratic and multidimensional experiences are represented as the complex constructs that they truly are. Grouping variables can be discovered through the application of classification algorithms such as hierarchical cluster analysis, latent profile analysis, or related methods. These approaches entail no *a priori* assumptions about what the relevant groups will be; instead, they identify sub-groups of children who have had similar experiences to one another, but different experiences than other sub-groups. A virtue of this approach is that it creates groups that are more internally homogeneous while also reflecting the reality that DHH children’s experiences with input are frequently multidimensional. Crucially, this approach can also accommodate information about the extent to which DHH children have lacked access to any form of input. There is of course no guarantee that the resulting profiles will cover the entire parameter space: however, this too turns out to be a virtue, in that it draws attention to areas of the parameter space that are not yet represented in the dataset and therefore potentially worth exploring.

### Dose-Response Functions

In healthy adults seeking relief from headache pain, the recommended dosage of aspirin is 300–600 mg every 4–6 hours. If someone takes only 100 mg a day and finds that their headache persists, they are not justified in concluding that aspirin is ineffective at relieving their headache pain. Meanwhile, if someone is taking 600 mg every 4 hours and the headache persists, then they would be justified in concluding that they might benefit from exploring other medications. The same reasoning applies to the relationship between language input and language outcomes. If Language A has constituted only 10% of a child’s input, it would be unsurprising to find that the child has not mastered Language A. But it would also be unjustified that therefore Language A does not benefit the child: rather, it has not been given a reasonable chance to succeed. However, if Language A has constituted upward of 60% of the child’s input and the child is not showing age-appropriate language skills, then it does stand to reason that the child -and others like them- may derive greater benefit from other types of input. It would also be important to determine whether the dose-response function is different for these children. For instance, it is possible that some DHH children would respond well to Language A, but only if it constitutes 85% or more of their input. It is also possible that even at this level, DHH children would still struggle to master Language A.

Unfortunately, extant research provides essentially no information about the dose-response relationship for various types of language input. One justifiable reason for this is the multidimensional nature of DHH children’s experiences, as described above: there may not be a monotonic relationship between amount of Language A and outcomes in Language A, because different types of input that are Not-A might have different effects. This is the primary justification for treating language input as a categorical rather than continuous predictor, provided that the levels of the categorical variable themselves represent multidimensional values.

More problematic than the absence of this dose-response information is the notion -implicit or explicit- that this information is in fact already known. This notion can surface in many forms. For example, an ASL advocate might promise a hearing family that their child can master ASL even if the primary source of ASL input is parents who are themselves novice learners. Or, an LSL advocate might counsel a family that signing is going to hurt their child’s chances of developing spoken language. Empirical evidence exists that is consistent with both of these claims (e.g., Percy-Smith et al., 2010; Allen, 2015; Henner et al., 2016; Geers et al., 2017); however, it is important to recognize that such studies occupy only one individual point somewhere along the broader dose-response function. As such, they cannot appropriately be generalized to other points along the continuum; unfortunately, such overgeneralizations appear to be commonplace. There does not appear to be any research that thoroughly documents the nature of the dose-response function between language input and language outcomes in DHH children. A major reason for this is the historical lack of methods for adequately characterizing language input. Developing and implementing such methods is therefore crucial to the goal of addressing questions such as the priorities identified by the CDC above. If language outcomes are measured but language input is not, how are we ever to know what kinds of input result in the best outcomes?

## Conclusion

Typically, language assessment focuses on language outcomes. As Moeller and Tomblin (2015) note, this is in part a reflection of theoretical traditions in which variation in linguistic input was thought to play only a minor or peripheral role in language acquisition. It is also a reflection of the tendency, at least in the United States, to treat white, middle class, monolingual children with no disabilities as the default standard to which all other children should be compared. Because such children have largely homogeneous distributions of language input, describing the child’s language input was not historically considered essential for understanding language outcomes. More recent work with culturally- and linguistically diverse populations has drawn the field’s attention to the importance of these factors, and to the associated drawbacks of relying too much on standardized assessments in clinical practice. Unfortunately, clinical work at the individual level has not always translated these concepts into practice in the most appropriate ways. Meanwhile, work at the population level has little recourse except to rely on the results of standardized tests, and as such is especially dependent on having information about children’s experiences with input in order to reach appropriate interpretations.

At the individual level, it would certainly be a mistake to not consider the child’s input at all, but it would also be a mistake to summarily dismiss all measures whose norms are derived from typically developing monolinguals. First, DHH children whose only language is English (whether LSL-only or in combination with English-based signing systems^5^) *are* in fact monolinguals: reduced knowledge of English in these children is not compensated by the presence of knowledge in another language. Likewise, it may be unsurprising to find that the mean of a sample of DHH children is likely to be significantly below the expected norm on standardized measures of spoken English, but paying attention to those children’s cumulative experience with input can help to discriminate whether this difference is unsurprising and unimportant, unsurprising but important, or perhaps even surprising and important (in a good way or a bad way). A DHH child can be showing progress toward or even achieving their IFSP goals while also still experiencing a significant language delay. Even in children who are showing good progress (e.g., making one year’s growth in one year’s time), the presence of a language delay can still have serious consequences for the child’s cognitive and social-emotional development, school readiness, and academic success. Therefore, intervention plans should look for strategies that are most likely to allow the child to make more than one year’s progress in one year’s time.

Paying attention to the input can also be a part of setting and tracking individualized goals, especially when there is reason to believe that changes in the child’s input would help them achieve their desired outcomes. There has been a lack of good methods for characterizing DHH children’s cumulative experience with linguistic input, but new tools are now becoming available that will facilitate these efforts. De Anda and Hall (in prep) provide a practical tutorial in using one such tool; it is hoped that other such tools and trainings will become available as the importance of considering the input becomes more widely appreciated. The present manuscript is offered in part to motivate the development of more resources and tools along these lines.

At the population level, tracking language outcomes without appropriately tracking cumulative language input risks yielding incomplete or even misleading information about upstream strategies that can minimize the adverse consequences of hearing loss. Relying on “communication mode” has now been shown to be deeply flawed, for a number of reasons: there is considerable diversity within children being raised with listening and spoken language (since language *access* is highly variable even within this group) and also within children whose experience includes access to various other forms of communication (e.g., not only variability in auditory access to spoken input, but also variability in the type of manual communication they use, and in the relative distribution of this input over a given period of time). Traditional top-down approaches to creating grouping variables are highly limited in their ability to accurately capture the complex and multidimensional aspects of DHH children’s experiences with linguistic input. Instead, bottom-up approaches using various classification algorithms have more potential to reveal insights about strategies that most consistently yield desirable language outcomes. Likewise, bearing in mind dose-response relationships between language input and language outcomes will be necessary in order to avoid prematurely dismissing certain types of communication as ineffective when in reality the dosage may have been too small to have had any appreciable impact. There is of course no guarantee that increasing the “dosage” would necessarily yield more favorable outcomes, and it is understandable that clinicians are reluctant to recommend strategies that remain empirically unproven. However, this also creates a self-fulfilling prophecy: without families who choose to pursue those strategies, crucial data will remain unavailable. This makes it all the more important that when families do pursue lesser-trod paths, public health systems are poised to capture that information in a way that is amenable to investigating natural variation in dose-response relationships, thereby beginning to build more of an evidence base to inform clinical recommendations for future generations. This is only possible if our approach to assessment considers not only the outcomes, but the cumulative input as well.

## Author Contributions

The author confirms being the sole contributor of this work and has approved it for publication.

## Conflict of Interest

The author declares that the research was conducted in the absence of any commercial or financial relationships that could be construed as a potential conflict of interest.

## Footnotes

1 This manuscript considers a language disorder to be a condition that would have compromised a child’s ability to acquire a language even in the presence of plentiful and accessible high-quality input.

2 Results are being presented as standard scores for convenience; it is assumed that a thorough evaluation would not rely entirely on standardized assessments.

3 Children with unilateral hearing loss were not included in these studies.

4 At the individual level, disagreements on success may also stem from the fact that hearing, speech, and language all have different standards of success, but parents may not fully grasp these distinctions.

5 Just as learning how to express English in Morse code, Braille, or semaphore does not make someone bilingual, neither does learning how to express English in cued or signed forms.
